# Where Is the Rural Creative Class? A Systematic Literature Review About Creative Industries in Low-Density Areas

**DOI:** 10.1007/s13132-023-01341-6

**Published:** 2023-05-01

**Authors:** Sofia R. Silva, Carla S. E. Marques, Anderson R. Galvão

**Affiliations:** 1 CETRAD Research Center & University of Trás-os-Montes e Alto Douro, Quinta de Prados, 5000-801 Vila Real, Portugal; 2 ESTG, Polytechnic of Porto, Rua Do Curral, 4610-156 Felgueiras, Portugal

**Keywords:** Creative industries, Regional development, Remote areas, Systematic literature review, Bibliometric literature review

## Abstract

Research in creative industries is mostly focused on urban and metropolitan areas. However, various authors have approached the creative industries from a regional, rural, or remote point of view. The objective of this study is to map and analyze research on creative industries in low-density areas and to identify the main theories and current and future trends within this theme. For data collection, only articles published in the Web of Science and SCOPUS databases were used, from which a set of 152 documents was obtained. For this study, we used R Bibliometrix software to assist in result analysis and VOSViewer software, to create a reference co-citation’s map, which allowed us to identify three clusters, whose themes we analyzed in detail. The results allowed to show (i) an increase in investigations into creative industries in low-density areas in recent years, (ii) the main journals and authors that have contributed the most to this theme, (iii) the identification of the theories most used in these studies, and (iv) finally, the identification of three clusters: remoteness and place relations with the creative industries (cluster 1), critical perspectives and the spatial distribution of talent (cluster 2), and cultural policies and the genesis of the creative class (cluster 3). This study contributes to mapping and critically summarizing the existing literature linking the creative industries and low-density areas. In addition, the study made it possible to identify current and future trends in order to enhance new lines of investigation.

## Introduction

Economies in developed countries have changed from economies based on material goods to the commercialization of intellectual property (Colapinto & Porlezza, 2012), and in the last two decades, the creative industries have gained relevance on public policies (Bell & Jayne, 2010; Evans, 2009; Hall, 2000; Peck, 2005). What we consider as “creative industries” today is highly influenced by the work of Richard Florida, in particular by the publishing of *The Rise of the Creative Class* (Florida, 2002a). As we will realize, there are other synonyms and delimitations of “creative industries,” but we can briefly define them as a group of economic activities, many of which have the potential to generate innovation and employment, in particular through intellectual property, whose aim is the production, reproduction, promotion, distribution, or commercialization of goods, services, and activities of content derived from cultural, artistic, or heritage origin or related to education or management ( European Parliament, 2021).

This theoretical influence of Florida’s creative class, connected to the creative city theory (Landry, 2000; Yencken, 1988) and to the empirical studies that, over time, linked the creative industries to the agglomeration economy (Jayne, 2005), added to an urban bias on the research about creative industries: a bias to favor urban and metropolitan areas as ideal and a natural habitat for creativity (Bell & Jayne, 2010; Townsend et al., 2017).

As a result, it was only later that low-density areas started to become a regular concern on behalf of researchers (Bell & Jayne, 2010). Another consequence of this bias and its acknowledgment is that initial approaches are based not on low-density areas themselves but critical perspectives about Florida’s work and the emphasis on urban areas (Markusen, 2006; Peck, 2005). Other authors would add empirical studies that show equal importance of the creative industries for regional, rural, and remote development when compared with cities (Abisuga Oyekunle & Sirayi, 2018; Andres & Chapain, 2013; Baum et al., 2009; Chapain & Comunian, 2010; Correa-Quezada et al., 2018; Daniel, 2013; Fleischmann et al., 2017; Gibson et al., 2010; Slach et al., 2013).

Nonetheless, only a few literature reviews were made about creative industries as a vehicle for development, very few bibliometric reviews, and none specifically about creative industries in low-density areas. Other SLRs we found using bibliometrics focus on phenomenons (clustering and gentrification) and/or specific countries (China, Spain and Italy) (Basaraba, 2021; Fengbao et al., 2019; Lazzeretti et al., 2008) or are about creative and cultural industries as a concept (Cho et al., 2018). This SLR distinguishes itself from previous ones by applying bibliometric methods specifically to the intersection of creative industries and low-density territories, thus aiming to map existing studies on creative industries and low-density areas, to identify key trends, and to propose avenues for future investigations.

This SLR used 152 results from SCOPUS and Web of Science databases. We used R Bibliometrix software to analyze the results and VOSViewer software to draw co-citation clusters, which were analyzed in detail.

When studying any subject inside the area of creative industries, we realize that its basic meaning is not well established. Thus, this article begins by reviewing the concepts of “creative industries” and “low-density areas.” Afterward, we present our methodology in detail. Then, we characterize the results and make a systematic literature review, followed by the cluster’s analysis through the bibliometric method. Finally, we present future lines of research and conclusions.

## Conceptual Review

### Creative Industries

The terminology “creative industries” was created in countries with a tradition of culture support and funding, like the UK and Australia (Galloway & Dunlop, 2007; Flew & Cunningham, 2010), and most authors identify the popularization of creative industries as a product of Tony Blair’s New Labour government policies, in the UK, when the Creative Industries Task Force (CITF) began as an activity of the newly created DCMS (Department of Culture, Media and Sport) (Drake, 2003; Flew & Cunningham, 2010; Galloway & Dunlop, 2007). According to Flew and Cunningham (2010), the DCMS was responsible for four main contributions: (1) it positioned the creative industries as a central element of the post-industrial economy of the UK; (2) it continued the trends that had been manifesting in the cultural policies of the UK and Australia in previous years; (3) it connected the creative industries to economic growth and moved creativity away from the traditional “subsidized arts” perspective; and (4) it created a list of economic activities included in the creative industries, from artistic to technologic, and grouped them under the same name.

This synthetic “political creation” of the creative industries generated semantic confusion with the previous term, “cultural industries,” and many European governments, hesitant to use the new nomenclature of “creative industries,” opted for the continued use of “cultural industries,” even though they added new meanings to it. In the Nordic countries, on the other hand, the preference was for “creative economy” (Flew & Cunningham, 2010), and in South America and Spain, it is common to use the synonym “orange economy” (González & Annayeska, 2020; Ferreiro-Seoane et al, 2022).

The choice between “cultural industries” and “creative industries” has implications in the analysis of theories, industries, and the policies themselves (Galloway & Dunlop, 2007). The critical review of Flew and Cunningham (2010) highlights that this debate is political and is born out of the context of the creation of “creative industries,” whose association with neoliberalism creates inequalities in the access to culture and its perception as a public good. If cultural institutions and associations are required to have the same degree of competition, productivity, and economic growth as others in the creative sector, social and cultural values, which should be in balance, disappear. This conflict relates to Swyngedouw’s (2007) post-political city, which describes the contemporary neoliberal city as a space of growing inequality, where the wealthiest enjoy the city made stage—of big events and big projects. This is a deeply divided city, shaped by the desire to make cities more “competitive,” to attract investment, further and further away from state control, and to commodify the social spaces that exist. Creatives become, as individuals, the symbol of this potential economic development (Peck, 2005). In addition, the need for semantic separation also stems from the separation in policies created by the advent of the creative industries: on the one hand, there are creative economic policies to increase competitiveness, opportunity, and low state intervention; on the other hand, there are public culture policies to subsidy the arts and culture as a public good.

One of the criticisms leveled at *The Rise of the Creative Class* (Florida, 2002a) is exactly how it ignores those who are not “part of the group” and the consequences of the migration of such a group. This group tends to avoid the suburbs and spaces without identity, preferring the centers instead of the outskirts of the city, where it absorbs and replaces the organic culture of “interesting” places. The creative class makes these places their own, as a starting shot for the gentrification phenomenon (Peck, 2005).

It is consensual that the term “cultural industries” precedes creative industries. Adorno and Horkheimer coined the term “cultural industry” to underline the paradoxical connection between culture and industry (Drake, 2003; Garnham, 2005; Galloway & Dunlop, 2007). At the end of the twentieth century, the term underwent semantic mutations and was assimilated by the creative industries. While the term “culture” was progressively abandoned and considered elitist and exclusive, the term “creativity” started to be perceived as democratic and inclusive (Galloway & Dunlop, 2007).

Nowadays, “creative industries” is the most used terminology, even though there is no consensus about which activities should be included in them (Barandiaran-Irastorza et al., 2020; Drake, 2003; Miguel & Herrero-Prieto, 2020). ) In the 2000s, UNESCO in 2007 and UNCTAD wrote reports that added new data and importance to the creative industries globally, establishing a new baseline framework for the creative economy (Flew & Cunningham, 2010).

This continued use of different words and paradigms created what Galloway and Dunlop (2007) named terminology clutter in the research area. Drake (2003) writes that we should understand the definition of creative industries as a definitional continuum and not as a category whose borders and define. Still, the terminology clutter requires, in the context of scientific research, that we explain which criteria will be used to define “creative industries.”

In this review, the results were obtained from the combination of keywords “creative” with “industry,” “economy,” or “work”; therefore, we are going to use “creative industries,” the most common term in our results. Like Drake (2003), this choice was motivated by the creation of consistency and not by the preference of one term.

### Low-Density Areas

The characterization of an area as “low-density” comes from a variety of criteria and parameters. Bento et al. (2013) analyzed how the concept of the low-density area was used in European studies and which indicators were utilized to characterize those areas. Bento et al. (2013) divide these studies into four groups: (1) population density and dynamics, (2) levels of development and socio-economic dynamics, (3) urban–rural relations and accessibilities, and (4) multi-criteria studies. From these different profiles, the authors define a “low-density area” as: “(…) a markedly rural and climatically severe territory, with an aging, losing, sparse and dispersed population, functionally peripheral and with decreasing accessibility to the main services and public goods, economically marginal and dependent on subsistence agriculture and/or social subsidies” (Bento et al., 2013, p. 582, translated from Portuguese).

In Europe, it is most frequent the use of OCDE’s characterization of low-density areas, established in 1994 (Bento et al., 2013). In Portugal, the semantic delimitation of low-density regions was defined in 2015 by Portugal 2020 Interministerial Coordination Commission (ICC) from a group of criteria: population density, geography, social and economic characteristics, accessibilities, demography, and settlements. In this classification suggested by ICC, of the total of 278 municipalities in mainland Portugal, 165 are classified as low-density territories.

The term “low-density” is consensual to all studies and the use of synonyms is frequent. Among them are “sparsely populated areas,” “less favored areas,” “peripheral areas,” or “under-populated areas” (Dubois et al., 2017; Le Tourneau, 2020; Pato, 2020). In this review, we chose “low-density regions” because it is the most common term in Portuguese classifications and whose definition, as presented by Bento et al. (2013), matches what we intend to mean, although the search for documents required other keywords, as explained in the next section.

## Methodology

“Remote places can sometimes provide a better CCI environment than major cities” is one of the five main research themes in creative industries, according to Cho et al. (2018) bibliometric review, but there is still to be made the mapping of this subject. Thus, our goals are to make a literature review about creative industries in low-density areas, to provide a global perspective on the subject, to identify the main theories, and to suggest future lines of research. This research is important because it opens new possibilities for low-density areas to be more competitive and challenge territorial imbalances through the attraction of creative and cultural industries.

To achieve these goals, we chose a hybrid literature review (Paul & Criado, 2020), combining both the bibliometric and the systematic types of review. The bibliometric method allows us to have a bird’s view through a quantitative approach (Paul & Criado, 2020; Zupic & Čater, 2015) and brings novelty to our study, since, until now, the bibliometric method was only scarce to the area creative industries (Lazzeretti et al., 2008). The systematic method, on the other hand, allows us to synthesize, specify, and compare results, most influential authors, methods, theories, and themes, while minimizing bias and ensuring the replicability of our results (Grant & Booth, 2009; Snyder, 2019). This approach is consistent with the one adopted by Rêgo et al. (2021).

**Fig. 1:**
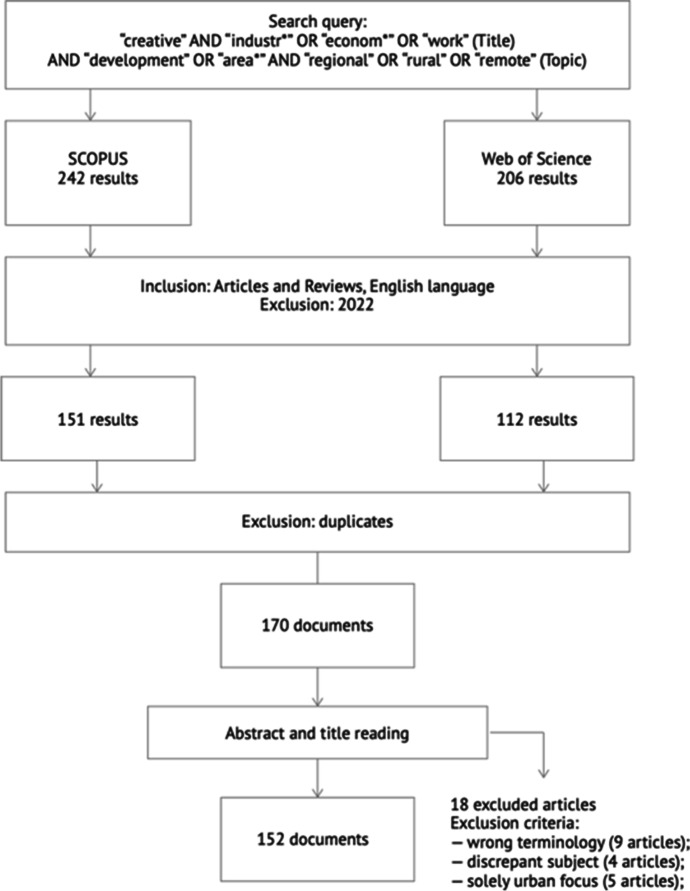
Steps for bibliographic search

The bibliographic search, whose steps are described in Fig. 1, was limited to SCOPUS (Elsevier) and Web of Science (Thomsson/Reuter) databases as a way of including the highest number possible of relevant results. These are databases of scientific research on various subjects related to creative industries, including management, social sciences, and culture. The plurality of subjects is essential due to the interdisciplinarity of the creative industries’ research subject. Both databases are internationally recognized and frequently used by the scientific community (Zupic & Čater, 2015; Paul & Criado, 2020).

As suggested by Xiao and Watson (2019, p.12), our keywords come from the research question “What has been studied about creative industries in low-density areas?”. Starting from the terms “creative industry” and “low-density areas/regions,” we defined the following search query: “creative” AND “industr*” OR “econom*” OR “work” in the title, together with “development” OR “area*” AND “regional” OR “rural” OR “remote” in the topic (abstract, title or keywords).

As presented before, the definition of creative industries is not consensual (Barandiaran-Irastorza et al., 2020; Drake, 2003; Miguel & Herrero-Prieto, 2020) and requires researchers to choose which terms to use. In this review, we opted for “creative industry,” complemented by “creative economy” and “creative work” as synonyms to ensure that our search was not limited by semantic or context incompatibilities (Pintilii et al., 2017; Schulte-Holthaus, 2018). We excluded the term “cultural industry” due to the need to narrow the results and focus this work specifically on the creative industry and its economic impact, related to the development and growth of rural or remote regions. This exercise of inclusion and exclusion of synonyms went through several tests to guarantee a balance between the degree of exhaustion and the degree of precision between the keywords (Xiao & Watson, 2019, p.12).

Concerning the low-density areas, after experimenting with the terms “low-density,” “sparsely populated,” and “peripheral areas,” which did not return any results or which results were irrelevant, we opted to use the keywords “development” and “area*” with “regional,” “rural,” or “remote” characteristics as synonyms of the low-density areas we want to focus.

After using the search query in both databases, 242 results were obtained in SCOPUS and 206 results in Web of Science. In a first step, all articles from the current year were excluded, as this search was conducted on March 1, 2022, and the data for the current year is not complete. Only papers in English were included, as this is the main language in the international scientific community and as a guarantee that all results would be readable in full. We limited the search to “article” and “literature review” type documents because of the easier access to the documents and because both articles and reviews tend to be more rigorous documents. After applying these three criteria, we obtained 151 results in SCOPUS and 112 in Web of Science, for a total of 263 results. After cross-referencing the databases and eliminating the repeats, we obtained a final result of 170 documents.

Of these 170 documents, we excluded 18 in the second phase, after reading titles and abstracts, according to the following criteria: (1) 9 articles were excluded because the keywords were being wrongly applied since we used the keywords “creative,” “industry,” “economy,” and “work” separately, and some results were using compound terms like “creative destruction,” “creative recovery,” or “creative social work,” all concepts that are not relevant to this research; (2) we excluded 4 articles that, although the terms are being correctly used, the global subject of the article was discrepant from ours, like articles whose theme was intellectual property or healthcare in the digital age; (3) 5 articles were excluded because their geographic focus was metropolitan areas and their goal was to analyze the growth and impact of creative industries in large urban areas. We kept all other articles mentioning “city,” “urban,” or “metropolitan” because, besides focusing on those areas, they also presented a regional and wider approach. After this second phase, we reached our final 152 results, from which 148 were classified as articles and 4 as literature reviews.

After establishing this final body of documents, the results were exported in two BibTex files. Using R Studio software (version 1.4.1106 for macOS, March 2022), we confirmed the elimination of duplicates and joined both files in a single database, which we verified to ensure that authors and keywords did not have inconsistencies or unnecessary repetitions. For example, the keywords creative industries (plural) and creative industry (singular) were homogenized for creative industries. For this study, we used R Bibliometrix software (version 3.0) to assist in results analysis and VOSViewer software (version 1.6.15), to create a reference co-citation map, which allowed us to identify three clusters, whose themes we will analyze in detail.

## Results

### Result Characterization

The urban bias in creative industry research (Bell & Jayne, 2010; Townsend et al., 2017) results in an under-representation of low-density areas. This under-representation is evident in our results’ dates, of which the oldest result is a single article from 2001 (Feldman, 2001, Towards the post-university: centers of higher learning and creative spaces as economic development and social change agents) followed by others from 2005, even though we did not set any date restriction.

**Fig. 2:**
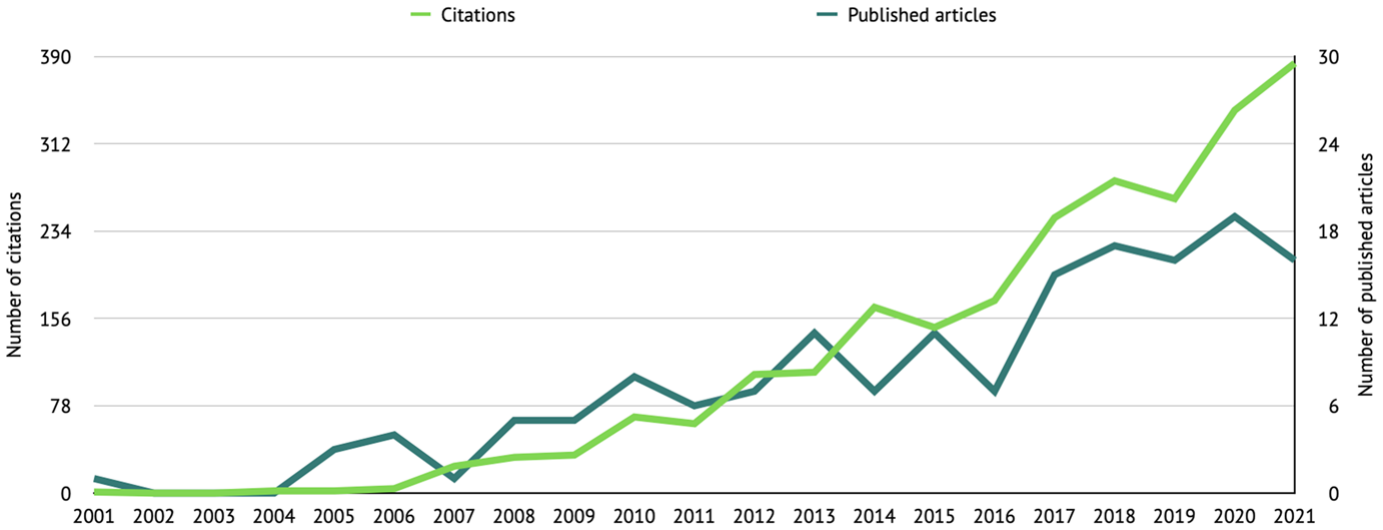
Evolution of published articles and citations by year (2001–2021)

In Fig. 2, we can see that the number of articles published since 2005 (*n* = 3) and until 2021 (*n* = 16) has been increasing and that close to half of the results (52.5%) were published in the last 4 years. The number of published articles grew significantly in 2017 and has been higher than 15 articles per year ever since. More meaningful than this growth is the increase in results’ citations from 2005 (*n* = 2) to 2021 (*n* = 475), which reflects a growing interest in the subject of creative industries in low-density areas.

**Fig. 3:**
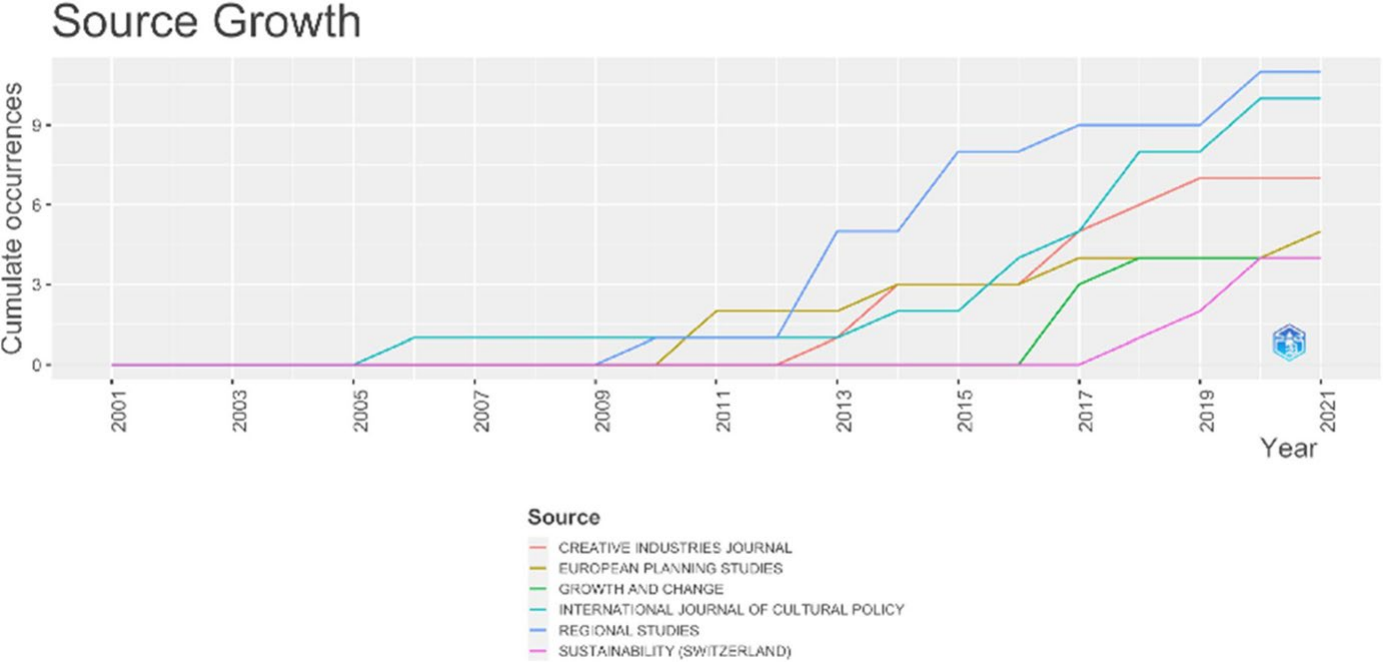
Top 5 journal dynamics per year

The research about creative industries crosses a wide range of subjects; thus, when we focus its relation with low-density areas, it is expected that this multidisciplinarity is kept. So, with this literature review, we seek to be as inclusive as possible, to not restrict areas of research, and to include all areas of the SSCI (Social Sciences Citation Index). The results obtained encompass 109 different journals, from which 5 have 5 or more published articles. Of the top 5 journals published on the subject (Fig. 3), 3 are journals of interdisciplinary research: *International Journal of Cultural Policy*, *Regional Studies*, and *Creative Industries Journal*.

Our research used as keywords “creative industry,” “creative economy,” and “creative work,” so the documents found reply primarily to that criteria; however, this choice did not eliminate other keywords and synonyms like “creative class,” “cultural industries,” “cultural policies,” “cultural economy,” “creative clusters,” or “creativity.” This variety of keywords shows that even limiting our initial query, the terminology clutter (Galloway & Dunlop, 2007) is evident.

**Fig. 4:**
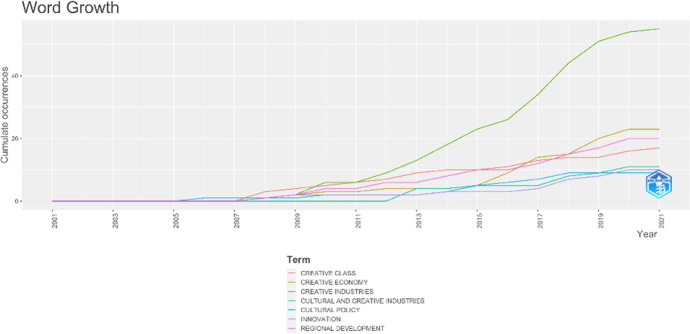
Author’s keyword dynamics per year

We can observe in Fig. 4 how the 7 most used keywords have evolved over the last two decades (2001–2021): creative industries (total = 55), creative economy (total = 23), regional development (total = 20), creative class (total = 17), innovation (total = 10), cultural and creative industries (total = 11), and cultural policy (total = 9).

**Fig. 5:**
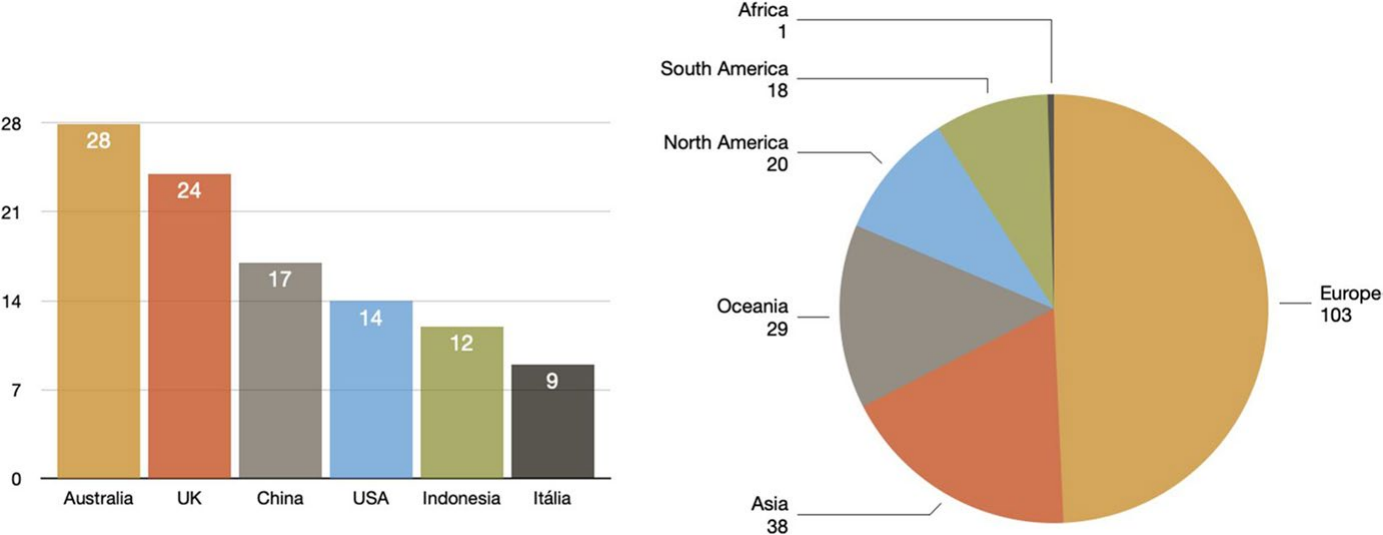
Top 6 countries that publish on the subject and number of articles by continent

The documents obtained are from 35 different countries. In Fig. 5, which presents the 6 countries with the most published articles, we can see that Australia has the most published articles (*n* = 28), followed by the UK (*n* = 26), China (*n* = 17), the USA (*n* = 14), Indonesia (*n* = 12), and Italy (*n* = 9).

When the analysis is done by continents (Fig. 5), Europe (*n* = 103) stands out first, followed by Asia (*n* = 38) and Oceania (*n* = 29). It is interesting to compare these results with those obtained by Cho et al. (2018) in their bibliometric review on the general topic of creative industries, where the USA comes first (*n* = 246), followed by the UK (*n* = 183) and Australia (*n* = 111), while the results of the top 7 countries in Asia are grouped (*n* = 46). This shows us that while the USA produces more scientific research on creative industries, countries such as the UK or Australia focus more on the topic of creative industries in regional context or remote areas; at the same time, the opposite is true for the proportion of articles from China and Indonesia, which stand out here in top places, while in the review by Cho et al. (2018), the countries of Asia do not have individual preponderance.

**Fig. 6:**
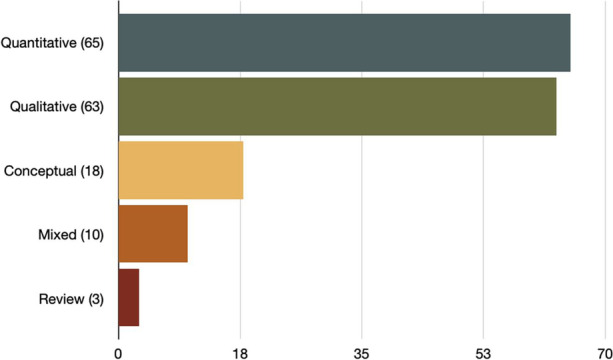
Results’ characterization by methods used

From the classification of the 152 results as to their methodology (Fig. 6), 138 are empirical studies, of which 65 follow quantitative methods, 63 apply qualitative methods, and 10 use mixed methods. Of the remainder, 18 are conceptual articles, and 3 are literature reviews. From these numbers, we deduce that there is a balance of methods in empirical studies and that literature reviews are the least frequent type of publication. The 3 literature reviews are divided into 2 bibliometric and systematic reviews and 1 narrative review (Xiao & Watson, 2019). Due to being scarce but important articles for the contextualization and relevance of our research, we further analyze each one in Table 1.

**Table 1:** Summary of literature reviews

Article	Method	Sources	Summary
Duxbury (2020). Culture and creative work in rural and remote areas: An emerging international conversation. *International Journal of Cultural Policy, 1–15*	Narrative review	Academic and gray literature	Identifies the main lines of research, main journals, and the recent evolution of research on cultural and creative work in remote areas
Cho et al. (2018). What are the concerns? Looking back on 15 years of research in cultural and creative industries. *International Journal of Cultural Policy*, *24(1)*, 25–44	Bibliometric review	Web of Science	Review about the global subject of creative industries. It analyzes 1002 documents, between 1997 and 2012. It identifies the main themes, one of which is the relation with remote areas
Lazzeretti et al. (2008). Do creative industries cluster? Mapping creative local production systems in Italy and Spain. *Industry and Innovation, 15(5),* 549–567	Bibliometric review	Web of Science	Focused on “creative economy” and its authors’ network. This review analyzes co-citations and the intellectual network of authors. It uses 941 documents, between 1998 and 2013

Of the total of results, 37 articles mention theories; of those, 34 are empirical studies that follow a quantitative methodology. Of the top 5 mentioned theories (Table 2), we highlight the creative class theory, mentioned by 27 articles.

**Table 2:** Most mentioned theories

Theory	Author	Year	Ment	Summary	Articles that mention the theory
Creative class	Richard Florida	2002	27	The creative class, as defined by Florida, is a set of highly trained and innovative professionals, capable of finding solutions to contemporary problems, whose presence is crucial to economic development, and whose migration between territories responds to Florida’s 3 T’s theory	Zhao et al. (2020); Audretsch and Belitski (2021); Duxbury (2020); Daniel (2014); Fazlagić and Skikiewicz (2019); Tiruneh et al. (2021); Mossig (2011); Bell and Jayne (2010); Tiruneh (2014); Currid-Halkett and Stolarick (2013); Hatcher et al. (2011); Batabyal and Nijkamp (2011); Ström and Nelson (2010); Baum et al. (2009); Donegan et al. (2008); Petrov (2008); Stam et al. (2008); Batabyal and Yoo (2018); Sternberg (2012)
Human capital	Gary S. Becker	1964	13	Human capital is the set of talents and skills of the inhabitants of a given location, acquired through education and experience. The appropriation and exploitation of these talents results in economic growth	Zhao et al. (2020); Ženka and Slach (2018); Ochoa and Ramírez (2018); Tiruneh et al. (2021); Lengyel and Ságvári (2011); Petrov and Cavin (2013); Donegan et al. (2008); Sternberg (2012)
Creative city	David Yencken	1988	6	This theory holds that a creative city is one that promotes creativity among its citizens and is emotionally satisfying to those who live there, as well as being an efficient and just city	Daniel (2014); Bell and Jayne (2010); Petrov (2008); Bagheri Kashkouli et al. (2018)
Creative capital	Richard Florida	2002	3	Formulated by Florida from the human capital theory, it differs from the latter by recognizing a specific group of human capital as a factor of economic growth. Creative capital is the economic power of the creative class and, therefore, the two theories are closely linked	Batabyal and Yoo (2018); Batabyal and Nijkamp (2011); Petrov and Cavin (2013)
Three T’s theory	Richard Florida	2002	2	Advocated by Florida as a recipe for economic growth in the twenty-first century. The three T’s stand for technology, talent, and tolerance. Technology being the most obvious, talent what cities need to retain (linked to creative capital), and tolerance the diversity that the creative class seeks and values in a place	Sternberg (2012); Wu and Li (2018)

This theory was presented by Richard Florid in his book *The Rise of the Creative Class: And How It’s Transforming Work, Leisure, Community and Everyday Life*, published in 2002. Florida argues that like in the Industrial Revolution, natural resources were fundamental to fuel the creation of great factory cities; in the twenty-first, the most valuable resource for economic growth is creativity, which is manifested by the class of professionals he calls the “creative class.” This is a highly educated class, with skills that allow them to solve current problems and actively look for new ones that will need solving. They are not only arts and media professionals (the areas usually understood as “creative” in the creative industries) but also from other areas of the knowledge-based economy, like engineering, healthcare, education, research, programming, science, or the law. One of the key points of Florida’s thesis is that this special class is attracted to places with high levels of tolerance, diversity, and amenities, which opposes the traditional view of the attractiveness of places by wealth and employment (Florida, 2002).

The creative class theory quickly became popular and caused a large number of cities to start investing in creative and cultural policies to capture the interest of this new class, sometimes uncritically (Peck, 2005; Garnham, 2005). The creative class theory defines 2 of our 3 co-citation clusters, which will be analyzed in the next part of this review.

**Fig. 7:**
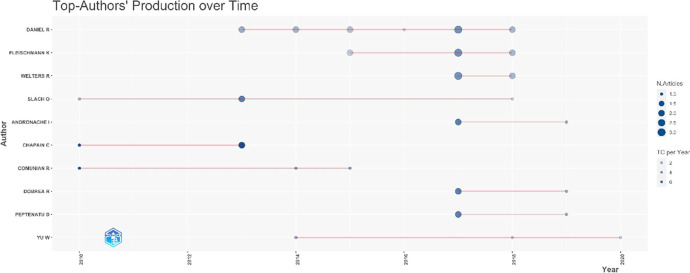
Top 10 authors’ production over time

Regarding authors, the author with the most articles in our results is Daniel (*n* = 12), followed by Fleischmann (*n* = 7), Welters (*n* = 5), and Slach (*n* = 4). Of these, the first three authors are based in Australia and have published collaborative papers; their focus is on the economic and social impact of the creative industries at a regional level, as well as their networks and key players, particularly in northern Australian cities such as Darwin, Cairns, and Townsville, which are geographically distant from major Australian urban centers (Daniel, 2013). Slach et al. (2013), on the other hand, are authors from the Czech Republic and their published articles study the characterization of creative industries in that country from a spatial and geographical perspective. We notice in Fig. 7 that these 4 authors were last published in 2018 and none of these most prolific authors published in 2021 and that the author with the most recent publication is Yu (with 3 published articles, the last one in 2020), followed by Andronache (with 2 published articles, the last one in 2019).

Yu publishes on the agglomeration of creative industries in China, with links to traditional industry and entrepreneurship; Andronache collaborated on two articles, one of which characterizes the creative economy in Romania (Pintilii et al., 2017), while the other is a study using Sholl and Kolmogorov analyses to assess the sustainable development of the creative economy in the Bucharest region of Romania (Gruia et al., 2019).

The most cited article, with a total of 256 citations, is “Entrepreneurship, innovation, and industrial development: Geography and the creative field revisited” by Scott in 2006, published by the *Small Business Economics* journal. Table 3 lists the articles with over 70 citations, their methodology, and main conclusions.

**Table 3:** Most cited articles

Article	Cit	Objective	Methods	Area	Main conclusions
Scott (2006). Entrepreneurship, innovation and industrial development: geography and the creative field revisited. *Small Business Economics*, 26(1), 1–24	256	Scott aims to sketch a geographical theory of the creative field from the way it constructs the functions of entrepreneurship, innovation, and economic development in the new economy. His approach to the economy is global, from technologically intensive manufacturing to producers of purely symbolic output	Conceptual	Non-applicable	Scott argues that geography is not just a passive frame of reference but an ingredient in economic growth and development. The creative field cannot be understood as a set of independent variables but rather in terms of interdependent structures (such as labor-territory, production factors), with different results depending on the geography and the time in which they are applied
Bell and Jayne (2010). The creative countryside: Policy and practice in the UK rural cultural economy. *Journal of Rural Studies*, 26(3), 209–218	133	Beginning with the Shropshire case study, analyze emerging policy and research on rural creative industries	Mixed, 36 interviews and 2 focus groups + analysis of statistical data	Shropshire (UK)	Urban territory dominates the focus of policies for the creative industries set over the last decade in the UK; however, in more recent years, not only have lobbies for rural policies and the arts emerged, but some rural regions have been able to take advantage of existing creative policies to reshape their regional economies
Chapain and Comunian (2010). Enabling and inhibiting the creative economy: The role of the local and regional dimensions in England. *Regional Studies*, 44(6), 717–734	88	Analyze the discrepancy that exists between the development of the creative industries in London and other regions of England by exploring factors that contribute to or inhibit that development	Qualitative, 167 interviews	Birmingham and Newcastle-Gateshead (UK)	Stakeholders point to several factors that characterize their relationship with the place where they live, including logistical difficulties and advantages of those places. It is important to consider the exchanges that take place outside the regional borders of these cities
Stam et al. (2008). Creative industries in the Netherlands: Structure, development, innovativeness and effects on urban growth. *Geografiska Annaler: Series B, Human Geography*, 90(2), 119–132	83	Explore the effects of creative industries on innovation, development, industrial and spatial structure, and employment growth in cities, specifically in the Netherlands	Quantitative, SME surveys	Netherlands	For job creation in the regions, improving living conditions for the creative class is likely to be more effective than stimulating the creation of new companies in the creative industries. If the goal, however, is to stimulate innovation, it does make sense to promote the creation of new creative businesses. However, this study shows heterogeneity in creative businesses and therefore advises that instead of designing a less effective policy that stimulates all fields, policies should be designed specifically for specific fields in the creative industries
Bontje and Musterd (2009). Creative industries, creative class and competitiveness: Expert opinions critically appraised. *Geoforum*, 40(5), 843–852	72	Contribute to the discussion by critically analyzing the opinion of local experts on policies for the development of creative industries and on the impact of the development of creative knowledge regions on the social fabric and other aspects	Qualitative, 196 interviews	7 European cities and their regions (Amsterdam, Barcelona, Birmingham, Helsinki, Leipzig, Manchester, and Munich)	There are more similarities than differences between the programs and policies implemented in these 7 cities, not only because the cities observe each other, but because these strategies are developed from the same sources. One such source is Florida’s work that is sometimes perceived as a “magic formula” for rescuing cities, while there are a variety of parameters that can be taken into account for promoting a more diverse vision of urban economic development
Donegan et al. (2008)Which indicators explain metropolitan economic performance best? Traditional or creative class. *Journal of the American Planning Association*, 74(2), 180–195	71	By contrasting regional creative capacity with traditional factors of competitiveness, explore the relationships between the presence of a creative class and regional economic performance	Quantitative, comparative analysis multivariate regression model	Non-applicable	Using human capital and industry composition works as well or better than Florida’s three T’s to explain wage growth and job instability. Attracting the creative class does not replace traditional strategies such as investing in education, increasing worker training, creating in business, or expanding existing industries

### Cluster Analysis

**Fig. 8:**
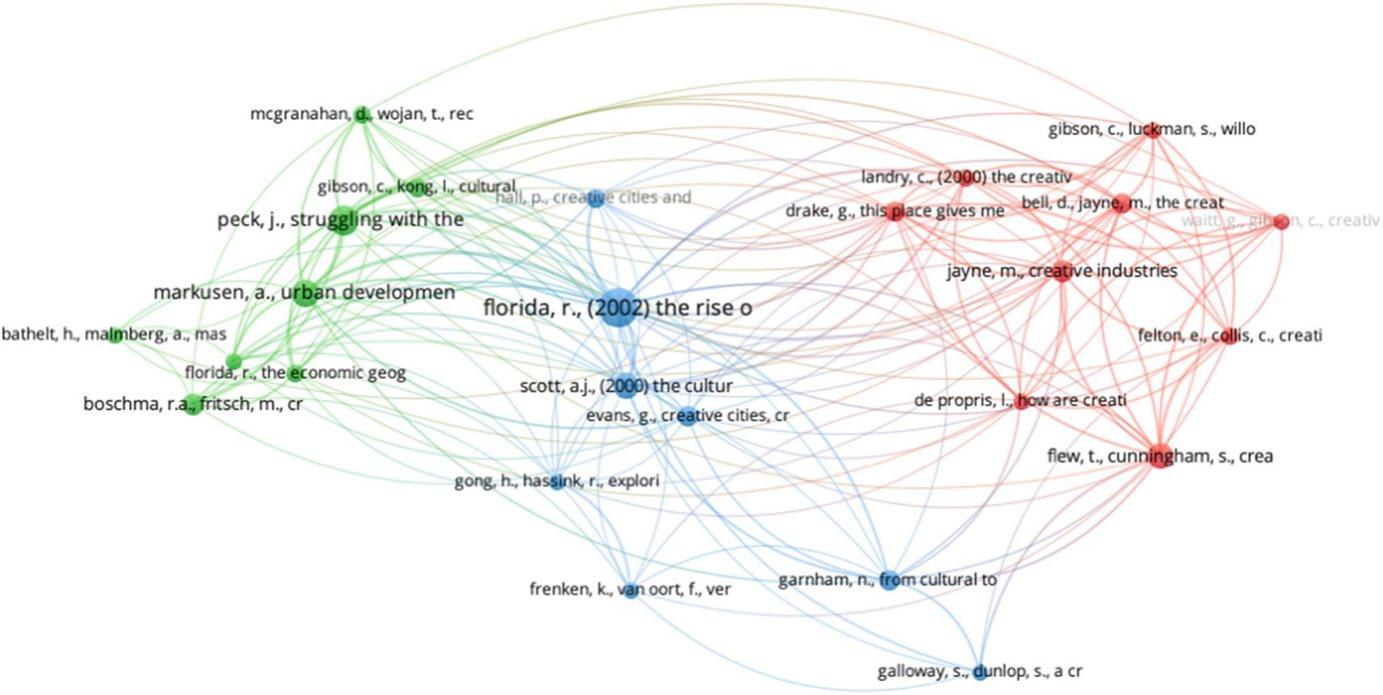
Co-cited reference map

To better understand the dominant themes in these 152 articles and how research on the topic is divided, we proceeded to analyze co-cited references using the VOSViewer program (Van Eck & Waltman, 2010). Figure 8 shows a map based on articles with a minimum of 5 co-citations, which resulted in a total of 25 documents, organized into 3 clusters.

The first cluster, with 9 articles (red, 36%), is the cluster most closely related to the theme of this review and focuses on the relationship of creative and cultural industries to places, in particular suburban, rural, and remote places. The second cluster, with 8 articles (green, 32%), gathers critical articles that focus on geographical dispersion and the agglomeration of talent; finally, the third cluster, with 8 articles (blue, 32%), focuses on cultural policies and the genesis of the creative class.

#### Cluster 1: Remoteness and Place Relations with the Creative Industries

This first cluster includes various studies about creative industries and places, in particular remote, rural, regional, and suburban places. It is the key cluster to understand the relationship between creative industries and low-density areas (Table 4).

**Table 4:** Top 4 most cited articles of cluster 1

Article	Co-citations	Objective	Methods	Main conclusions
Creative Industries after the first decade of debate (Flew & Cunningham, 2010)	12	Grouping critiques and perspectives around the creative industries and neoliberalism	Narrative review	Policies applied to the creative industries are distinguished from more traditional cultural policies by having three distinct characteristics: a focus on SMEs, an emphasis on the power of the Digital Age in transforming the producer–consumer model, and assigning public cultural institutions the role of incubators in the creative economy
Creative Industries: the regional dimension? (Jayne, 2005)	9	Critical analysis of the DCMS work and reports developed on the regional aspects of the creative industries in the UK	Conceptual	The enthusiasm for creative industries in big cities has not been followed up at the regional level. Creative industry policies are dominated by a cluster agenda, which distorts their true objectives
This place gives me space: place and creativity in the creative industries (Drake, 2003)	8	It investigates how workers in the creative industries (metalworking and digital sector) consider the characteristics of the place where they live to be a source of ideas, materials, and other elements for the creative process	Qualitative	Creativity is a collective process and is influenced by the place where the main activity takes place, both from the more classical perspective of inspiration and creative social networks, and because it brings economic, social, and family benefits
The creative countryside: policy and practice in the UK rural economy (Bell & Jayne, 2005)	8	Beginning with the Shropshire case study, analyze emerging policy and research on rural creative industries	Mixed	Urban territory dominates the focus of policies for the creative industries set over the last decade in the UK; however, in more recent years, not only have lobbies for rural policies and the arts emerged, but some rural regions have been able to take advantage of existing creative policies to reshape their regional economies

Flew and Cunningham (2010) draw the historiography of the definition of creative industries until its most consensual version, although never unanimous. In their analysis of cultural policies, the authors differentiate 4 types of geographic models: (1) an American model, where there is a clear separation between arts and culture from entertainment and copyright and where cultural policy is very localized; (2) a European model that highlights the cultural mission of the creative industries and promotes strategies for social inclusion and the common cultural benefit; (3) a variety of Asian models that emphasize the role of national social and cultural policies but use the creative industries as an opportunity of rebranding and export growth; (4) a variety of models from developing countries in South America, from South Africa, the Caribbean, and other places, where questions of cultural heritage, relief of poverty, and access to basic infrastructures overthrow a technocratic view of the creative industries as a natural product of the Age of Information.

Jayne (2005) critically assesses the work of the DCMS about the regional aspects of the creative industries in the UK and concludes that, although there’s an ongoing promotion of the creative industries and their economic relevance, the UK is not responding to that at a regional level. Jayne (2005) mentions the case of countries like Canada or Australia, who managed to implement policies that unite the cultural and the creative industries by separating the private and public/volunteer sectors, a problematic idea for the UK.

About the tendency of creative industries for agglomeration, Jayne (2005) refers that the inadequacy of policies at a regional level comes from the emphasis on the “cluster” and agglomerations, while the development of creative industries should respect the variety of place scales, like an ecosystem, from individual creative workers to small businesses, networks of businesses, to the larger clusters, in many geographic levels—city, region, country, and international.

Drake (2003) aims to comprehend the impact of place in the creative industries’ economic activities and how their agents relate to the place they live in. Scott (2000, cluster 3) establishes a close relationship between place and creativity, in the context of a cultural economy, suggesting that places can have specific characteristics that fuel creation and that places, where there is an agglomeration of creative industries, are, naturally, places that channel creativity (Drake, 2003). In his qualitative analysis of interviews, Drake (2003) concluded that for some interviewees:

“Locality is important (…) but not primarily because of its potential as a source of signs, ideas, and prompts. The attributes of the locality are perceived as important in terms of reduced costs, marketing advantages, social or family networks, familiarity or local infrastructure.” (Drake, 2003, p. 517).

His interviews with works from the metalworking and the digital sectors (two very different activities, on purpose) led Drake (2003) to define 4 types of effects of place over creative work: (1) the place as a resource, both visual (inspiration) and material; (2) the place as a stage for the social and cultural network; (3) the place whose reputation or traditions influence the outcome, and (4) the place where the community of the creative worker is.

#### Cluster 2: Critical Perspectives and the Spatial Distribution of Talent

In cluster 2, various critical perspectives are grouped, particularly directed to Florida’s creative class theory (Boschma & Fritsch, 2009; Markusen, 2006; Mcgranahan & Wojan, 2007; Peck, 2005). Although less expressive, there is also an article by Florida in this cluster (The Economic Geography of Talent, Florida, 2002b). The references grouped in this cluster allow us to understand the other side of the debate about cultural policy and how both the creative class and the creative city theories have flaws for the economic growth of places; in particular, they ignore the role of regions and fail to focus beyond the city as the desirable place for the creative class (Table 5).

**Table 5:** Top 4 most cited articles of cluster 2

Article	Co-citations	Objective	Methods	Main conclusions
Struggling with the Creative Class (Peck, 2005)	16	Critically counter the creative class theory	Conceptual	The popularity of Florida’s theory has inspired the creation of policies with little critical thought, based on flimsy data. The proliferation of these policies limits urban policy agendas
Urban development and the politics of a creative class (Markusen, 2006)	14	Critically analyze creative class theory from the case study of artists	Mixed	The author suggests that it is not the amenities but the investment that cities make in arts spaces and organizations, which attract the creative class
Creative class and regional growth: Empirical Evidence from Seven European Countries (Boschma & Fritsch, 2009)	9	Test some of Florida’s ideas in *The Rise of the Creative Class*, from regional data from seven European countries	Quantitative	The distribution of the creative class in Europe is uneven. The main factor for this distribution is the tolerance and openness of the territories, more than educational or health infrastructures
Cultural Economy: A critical Review (Gibson & Kong, 2005)	7	Review of the “cultural economy” from the most significant works of Adorno and Horkheimer (1977)	Critical review	Without definitive conclusions, they reaffirm the fluidity of the concept of cultural economy. They explore the idea of a “normative cultural economy,” which would delimit the concept but remove some of its complexity

In this cluster, we learn new perspectives about the creative class theory that amplify the subject from a critical point of view. Peck (2005) is the most critic of Florida’s (2002a) work, which he considers vague, although extremely popular. For Peck (2005), the exclusivity and elitism associated with the creative class have negative effects for the policies designed based on them and for those who are not included in the group—the creative class, for Peck (2005), feeds the inequality of places and the idea that labor instability is the “new freedom,” themes that, like the precariousness of many creative jobs, are ignored in Florida’s work.

One of the paradoxes of the creative class noted by both authors (Florida, 2002a; Peck, 2005) is its tendency to look for “genuine” places and, as a result, to flood those places, to be an agent for gentrification and, finally, to help destroy the originality and genuineness of the place.

The popular theory that the creative class would be the only path to take for economic growth in the twenty-first century led cities to enter a race for creativity. Peck (2005) criticizes the city ranking and the business model established by Florida around the consultancy and technical support to cities that wished to be more creative. Peck (2005) concludes that the idea of the creative class became so popular not because it is revolutionary, but because it is modest (p.760) because it fitted the neoliberal agenda already in motion.

McGranahan and Wojan (2007), Boschma and Fritsch (2009), and Markusen (2006) start from the creative class theory and extend its measuring indexes: McGranahan and Wojan (2007) exclude some of the economic activities originally considered by Florida (2022a) as part of the creative class, the use the Thinking Creative Index to measure precisely the presence of the creative class in rural areas. They argue that local economic growth happens through job creation and not merely by the migration of new creative works, even though job creation and the settlement of members of the creative class appear to have a causality relation.

Boschma and Fritsch (2009) apply the same indexes established by Florida (2002a) to the context of seven European countries (Denmark, England, and Whales, Finland, Germany, Netherlands, Norway, and Sweden) and present the distribution of Europe’s creative class, which, they conclude, is unbalanced. These authors attribute the unbalance to differences in the tolerance levels of regions, which, as they demonstrate, is Florida’s (2002) parameter with the most important effect.

Markusen (2006) criticized Florida’s (2002) theory from an element of the creative class, the artists, whose geographical distribution is conditioned by their personal preferences, job offers, and effects of the place in creativity. Markusen (2006) shows that infrastructures and places built for artists (like art centers, studio buildings, and small venues) are more effective in attracting and settling these creative works than the 3 T’s (tolerance, technology, and talent) argued by Florida (2002a).

About the distribution of creative industries in low-density areas, McGranahan and Wojan (2007) refer to a higher increase of creative population in areas with mountains, forests, or open spaces, where winter is sunnier (p. 208), which emphasizes the importance of natural amenities and weather for the rural creative class more than its urban counterparts. Other relevant characteristics pointed out by McGranahan and Wojan (2007) are as follows: (a) the high proportion of young adults with higher education in relation with the creative class growth, (b) the quality of life as a motive for the urban to rural migration, and (c) the tendency for the rural creative class to be older than the urban creative class and, therefore, to value the quality of schools instead of nightlife amenities, for example.

#### Cluster 3: Cultural Policies and the Genesis of the Creative Class

The third cluster leads us on the transition for a cultural economy, based on goods of high symbolic value and whose concerns are beyond mere survival (Galloway & Dunlop, 2007; Hall, 2000; Scott, 2000). This shift in the value of goods created various new economic activities that did not previously exist and whose income became relevant to economic growth (Hall, 2000); in the shift from the manufacturing economy to the information economy and finally to the cultural economy (Hall, 2000), culture came to be viewed as a highly valuable replacement for the factories of the past. This race to cultural policies led to the proliferation of terminologies (Garnham, 2005) and to the theorization of the creative class (Florida, 2002a) which, in its turn, was widely used by policymakers as a theoretical based for new initiatives, development projects, and policies for the cities of the new century (Table 6). This transition is summarized in the orange economy, which encompasses both the cultural economy and the creative industries and the areas that support it, and which, as shown by Ferreiro-Seoane et al. (2022), grows proportionally more than the economy as a whole.

**Table 6:** Top 4 most cited articles of cluster 3

Article	Co-citations	Objective	Methods	Main conclusions
*The Rise of the Creative Class* (Florida, 2002a)	28	Introducing the creative class and creativity as the essential elements for economic growth in the twenty-first century	Conceptual	According to Florida, the “creative class” is the main driver of economic growth. This group is attracted to places with high levels of tolerance, heterogeneity, and diversity, as well as places with infrastructure such as theaters, bars, museums, or restaurants. Florida’s focus is on the person rather than the business or industry. Jobs will follow people instead of people following jobs
*The Cultural Economy of Cities* (Scott, 2000)	12	Explore how the effects of production processes, with increasingly cultural outputs, make themselves felt in the growth and development of places	Conceptual	Given culture’s connection to identity and power, the increasing importance of the cultural economy raises political issues. Active and serious cultural policies are essential to control the economic destiny of places and, even more importantly, to reverse capitalist trends in cultural production
Creative Cities, Creative Spaces and Urban Policy (Evans, 2009)	8	From a survey, it presents the results of an international study on policies and strategies of the creative industries	Quantitative	These policies are sometimes implemented on very weak conceptual foundations, behind a tree, and it would be beneficial to consider the most distinctive aspects of each city (and its surrounding region)
From cultural to creative industries (Garnham, 2005)	8	Analyze the implications of the change in terminology (from cultural industries to creative industries) for arts and media policy and for research associated with the two terms	Qualitative	The terminological change serves to disguise political dilemmas and contradictions. The democratization of culture under the guise of creative industries calls into question the very support and subsidy for culture

According to Scott (2000), as we move to the twenty-first century, it seems to exist a convergency between cultural and economic development. At some point in time (around the creation of the DCMS, as we discussed before), there was a shift in terminology and, instead of talking about cultural economy and industries, the emphasis changed to creative economy and industries. Garnham (2005) argues that this shift in terminology is neither neutral nor casual and encompasses political and theoretical questions. Moreover, the term “creative industries” is more than a new version of “cultural industries” and it only makes sense in the context of this century’s policies (Garnham, 2005; Galloway & Dunlop, 2007). Historically, if there was a difference between policies for the arts and culture on one side, and policies for the media on the other, with the new advent of creativity, the two join in a hybrid that affects all sectors and are called both cultural and creative policy, depending on the context (Garnham, 2005). In this terminology clutter, the orange economy emerges as a possible umbrella term that encompasses and organizes the various definitions.

Various authors mention how implemented policies connect the creative industries to the need for cluster creation, either a business, technological, or industrial cluster (Scott, 2000; Garnham, 2005; Evans, 2009). The foundations of this connection come from the fact that creativity is not only a result of individual rumination but a consequence of many stimuli and interactions between different agents (Scott, 2000) and from the evidence that creative and cultural industries are more common in larger cities (Hall, 2000; Scott, 2000).

For cities that want to be more creative, talent is the most important resource (Hall, 2000): the constant renovation of talent is a key element for creative cities, which makes them, many times, very uncomfortable places to live in, where there is intellectual and social turbulence (Hall, 2000), in contrast with highly conservative and stable cities. Hall (2000) adds that, even though creativity seems to surface in places with high levels of innovation, this is not a direct causality and both time and luck also happen for cities.

Evans (2009) considers that measuring and classifying this new hybrid economy, whose base definition seems simple, is a challenge both for policymakers and researchers, and that is why indexes, rankings, and other systems are so compelling and quoted—they are an attempt to make the subject more palpable and conquer the inconsistencies in foundations and terminologies. The emphasis on indexes and parameters feeds the design and implementation of policies to improve results and measurements and therefore make cities even smarter and more creative (Evans, 2009). It is this context of the race to creative policies and attempts to understand the transition for the cultural economy that gives space for Florida (2002a) to theorize the creative class.

The creative class theory, as we discussed before, is one of the key concepts for the analysis of creative industries in low-density areas, although the original creative class prefers and chooses cities to settle. It is important to highlight that, contrary to the common perception of the creative industries, Florida (2002a) includes traditional knowledge workers in his creative class and does not limit it to arts, culture, and media.

#### Cluster Relations

The three clusters are interrelated. Cluster 3 leads us from the terminology challenges to the theorization of the creative class, cluster 2 questions that theory presents new points of view, and cluster 1 introduces rural, regional, and remote places as a perspective to consider.

Clusters 2 and 3 have a strong connection. Various articles from cluster 2 criticized the creative class theory, presented by the most relevant publication in our co-citation’s map, which belongs to cluster 3 (*The Rise of the Creative Class*, with 28 citations). Both clusters function as theoretical fundamentals for cluster 1, where geography and places gain ground.

To understand the subject of creative industries in low-density areas and how the general research in creative industries was self-critic and developed the need to expand its geographic focus, the group of these three clusters creates a theoretical and essential base, linearly leading us: beginning with the birth of the terminology and its challenges, up until the context for the creation of the creative class theory (cluster 3), followed by the popularization of creativity in public policy and its consequences, the growing urban bias, and the critic of both this trends (cluster 2) and finally the attempt to expand and apply scientific research to peripheral, rural, regional, and suburban territories (cluster 1).

## Future Lines of Research

This SLR gave us a birds’ view on the subject of creative industries in low-density areas; however, there are still areas of that subject that would benefit from further research.

Firstly, it would be important to draw the profile of the creative worker who moves to low-density areas. McGranahan and Wojan (2007) suggest that the rural creative class is older and looks for different characteristics in a place to settle. Hoey (2005) shows in his ethnographic work about internal migrations that the search for “a better quality of life” is the main reason for this move; Duxbury (2020, p. 6) refers to various authors who conclude that the migration of artists for smaller communities is due to high rents in larger cities and the search for a “different quality of life.” Defining the profile of the creative worker (and his business) who moved to low-density areas would be crucial not only to separate them from the urban creative class and its needs but to design policies specifically thought for the rural creative class (Bell & Jayne, 2010).

Also, in a further phase, following what was made by Townsend et al. (2017) with the study about the effects of broadband in rural Scotland, there is a need to research and comprehend the challenges the rural creative class faces when trying to remain and settle in the low-density areas, long term. Townsend et al. (2017) suggest that the lack of internet connection is one of the main ones, but there are others like isolation from the social network or even negative feedback from the local community.

With the various studies on the economic impact of creative industries, we can infer that, in areas with more fragile economies, this impact can be even more notorious for various reasons. Besides that, as demonstrated by Drake (2003), the place influences the creation and the outcomes of the creative industries. It will be relevant to study that exchange and understand what are the factual effects of the creative industries settling in a particular area, both for the territory and for the settlers.

The COVID-19 pandemic boosted remote work in many areas, including the creative industries, whose agents became even more dependent on wi-fi connection and less on place and geography. Has this change had any impact in motivating the migration of the creative industries?

Finally, the creative industries are connected to public policies. Various authors studied policies in a regional context (Bell & Jayne, 2010; McGranahan & Wojan, 2007), and we believe it will be relevant to make an SLR focused solely on the subject of policies and incentives to capture and settle creative industries in low-density areas. This work, together with the data obtained by studying the creative worker profile, his motivations, challenges, and behaviors, would allow us to design future lines for regional development based on the creative industries.

In summary, we suggest the following lines of research on the subject of creative industries in low-density areas:

- Characterize the profile and motivations of the creative worker who migrates to low-density areas.
- Understand the hurdles and challenges the creative industries face to stay in this areas.
- Understand the perception of local communities and the impact of creative industries from the natives’ perspective.
- Comprehend what type of symbiosis and mutations the creative industries generate and/or suffer when moving from urban to rural areas.
- Understand what was the effect of the COVID-19 pandemic and the popularization of remote work in the creative migration to low-density areas.
- Review what types of policies and incentives for creative migrations have been tried, specifically in low-density areas, who they targeted, and what were the results.

The promise of creativity as the twenty-first century’s raw material of choice has led many cities to bet on creative industries as a policy strategy for economic development; creative industries drive economic growth and are sources of innovation (Kanó et al, 2022). Low-density territories, with different concerns than metropolitan areas, closely follow this trend and imitate the main axes, running the risk of losing their distinctive characteristics as a territory (Bell & Jayne, 2010). In a country like Portugal, where 59.3% of the mainland municipalities are considered low-density territories, the creative industries may present themselves as an opportunity to combat the desertification of the interior (Bento et al, 2013). We should try to understand how.

## Conclusions

This SLR used data from the SCOPUS and Web of Science databases, with no time limits. From the analysis of co-citations, we identified 3 thematic clusters: remoteness and place relations with the creative industries (cluster 1), critical perspectives and the spatial distribution of talent (cluster 2), and cultural policies and the genesis of the creative class (cluster 3).

The results used in this review, as with any in the area of creative industries, are multidisciplinary and were published in a wide variety of indexed journals; however, this review shows the lack of other literature reviews on the subjects of creative industries in low-density areas; a flaw, we hoped, contributed to fill.

The research on creative industries in low-density areas shares some of the same challenges of the wider research on creative industries: the terminology clutter, the emphasis on agglomeration and clustering, and the debate around policies and their relation to culture. Like the cities of the twenty-first century, rural areas are affected by the same crisis and the same changes in paradigms, and the local policies lean on converging to the creative economy ideas (Bell & Jayne, 2010).

This study brings three important theoretical contributions to the existing literature. First, this study led to a survey and mapping of the main investigations on creative industries and low-density territories. Second, this study presents some current and future trends in this area, allowing it to be a starting point for further investigations. Finally, it contributed to the suggestion of future investigations based on the gaps that still exist in the literature. Regarding the practical implications, this SLR suggests that the relationship between creative industries and low-density areas is an emerging and important issue for regional development and that needs to have more attention from the governments, through specific policies to support this type of entrepreneurs.

In the course of this investigation, some limitations were found. The first limitation to take into account is subjectivity, despite having taken all precautions; this type of study always presents some subjectivity, both in the classification of articles and in the choice of keywords and the steps to be followed. Another limitation is related to the fact that book chapters, books, and conference articles were not included in the analyzed documents, since most of them are not easy to access.
